# Preparation and Application of Humidity-Adaptive Wooden Artifact Crack Consolidants Based on Lignin–Epoxy Acrylate

**DOI:** 10.3390/polym17101395

**Published:** 2025-05-19

**Authors:** Qijun Huang, Wangting Wu, Yingzhu Wang, Jianrui Zha

**Affiliations:** 1 Key Laboratory of Archaeomaterials and Conservation, Ministry of Education, Institute of Cultural Heritage and History of Science and Technology, University of Science and Technology Beijing, Beijing 100083, China; huangqj1117@126.com; 2 Capital Museum, Beijing 100045, China; wangyingzhu88@163.com

**Keywords:** wooden artifacts, crack reinforcement, hygroscopicity, material compatibility, epoxy acrylate

## Abstract

Due to compatibility issues between traditional reinforcing materials and the substrate of museum wooden artifacts, interface failure occurs after crack reinforcement, particularly under dry and wet cycling conditions. This significantly compromises the durability of reinforcement. To resolve this issue, dealkalized lignin was grafted onto epoxy acrylate (LEA) to synthesize a novel consolidant with both humidity responsiveness and mechanical compatibility. The resulting LEA exhibited excellent multilayer adsorption capability and demonstrated synchronous and uniform hygroscopic expansion behavior, closely matching that of archeological wood. DMA revealed that LEA2 has an elastic modulus of 261.58 MPa and a Poisson’s ratio of 0.35, comparable to artificially degraded wood, effectively mitigating interface stress caused by rigidity differences. Furthermore, LEA effictively reinforced micron-scale cracks while maintaining the original microstructure of the wooden artifact. This material provides a promising solution to the compatibility challenges of traditional consolidants under humidity fluctuations and offers a new approach for the stable preservation of museum wooden artifacts.

## 1. Introduction

Museum wooden artifacts constitute an important component of human cultural heritage. They encompass a wide range of types, including tools for production, living utensils, and means of transportation, serving as crucial evidence of history, society, and daily life in ancient times [1]. These artifacts are abundant in historical, artistic, and technological evidence and possess irreplaceable value for the study and preservation of cultural heritage [2]. However, these artifacts are confronted with various degradation phenomena due to environmental factors and material, including cracking, warping, insect infestation, mold growth, and salt efflorescence [3,4]. Cracking is a predominant and representative type of damage. It not only compromises the mechanical integrity of the artifact but also provides pore for moisture, pollutants, and microorganisms to infiltrate, thereby accelerating further degradation [5].

Environmental humidity fluctuation is a critical factor driving crack initiation and propagation in wooden artifacts. As typical hydrophilic porous materials, wooden artifacts exhibit significant sensitivity to ambient relative humidity (RH) variations [6]. When environmental humidity increases, water molecules penetrate and adsorb into cell walls through capillary action and surface adsorption mechanisms, inducing volumetric expansion of the artifact [7]. Conversely, a reduction in humidity results in moisture desorption, leading to shrinkage. Prolonged exposure to this repeated and uneven cycle of wetting and drying can result in stress accumulation within the artifact, especially in vulnerabilities areas. The accumulation of these stresses ultimately leads to crack propagation, warping, and even irreversible damage such as breakage [8]. Therefore, to inhibit the development of cracks in wooden cultural relics and improve mechanical stability, it is crucial to reinforce cracks to guarantee the long-term preservation of the artifacts.

The materials currently used for reinforcing wooden artifacts can be classified into three types: natural materials, synthetic resins, and composite materials. Natural materials, such as beeswax, paraffin, linseed oil, tung oil, and rosin, were widely used in early-stage conservation [9]. These materials have wettability and permeation properties that allow them to seal cracks, thereby enhancing the wood’s weather resistance properties [10]. Above them, synthetic resins have been widely used in crack repair, due to their excellent mechanical properties and stability. Epoxy resins demonstrate exceptional adhesive properties and strength, making them suitable for crack repair and the reinforcement of large wooden components, such as used in the restoration of wooden components in ancient buildings in Brazil and Spain [11,12,13]. Nevertheless, the irreversibility and high rigidity of epoxy resins constrain the application in the preservation of museum artifacts. Acrylic resins, such as Paraloid B-72, valued for their transparency, reversibility, and stability, are appropriate for reinforcing shallow cracks, though their durability under UV exposure still requires improvement [14,15,16]. In recent years, composite materials and the use of nanoconsolidants have also been applied in crack reinforcement research [17]. The microcrystalline cellulose/Paraloid B-72 system effectively enhances the hardness and structural support of the crack [18]. While these materials have improved the mechanical properties of wooden artifacts in the short term, the compatibility between the reinforcing materials and the wooden artifacts, especially the hygroscopicity mismatch, remains a key factor influencing the long-term stability of the reinforcement system.

The hygroscopicity mismatch between reinforcement materials and wooden artifacts primarily affects reinforcing efficacy in two ways, interface failure and strength incompatibility. Firstly, a hygroscopic mismatch between the consolidant and wood substrate results in divergent dimensional responses to humidity variations, causing stress accumulation at the interface. This may result in debonding, crack initiation, and potential crack propagation [19]. Secondly, the disparity in mechanical properties between the reinforcement material and wood, especially during wet–dry cycles, further amplifies the internal stress difference, increasing the risk of damage [20]. Therefore, achieving coordinated hygroscopic behavior and mechanical performance compatibility in crack repair materials is crucial for enhancing consolidation efficacy and ensuring the long-term stability of wooden artifacts.

Currently, the application of functionalized lignin materials in the reinforcement of wooden artifacts has gradually gained attention, showing great potential for sustainability and performance modulation [21]. As one of the most stable components among natural polymers, lignin is often retained even after the severe degradation of cellulose and hemicellulose [22]. As an important “residual framework” in archeological wood, its high reactivity provides a solid foundation for functionalization research. McHale et al. employed the in situ polymerization of polyisoeugenol to reinforce severely degraded timber components from the Oseberg shipwreck. This material does not reinforce the cell cavities, preserving re-treatment space, and embodies the concept of “reversible” conservation [23]. The same research group developed a polyurethane foam material based on lignin and tannins, which possesses excellent thermal properties and an original cell structure. It provides effective support while preserving wood porosity [24]. Furthermore, lignin, rich in phenolic hydroxyl groups, can replace phenol in the synthesis of phenolic resins (Lignin-PF). It is used in the coating, bonding, and repair of wooden artifacts. Lignin-PF demonstrates good mechanical properties and environmental adaptability, making it an important direction for future research on green reinforcement materials [25,26]. Functionalized lignin materials exhibit excellent physicochemical properties and can react with multiple functional groups, enabling customized designs to meet the specific reinforcement needs of artifacts, thereby enhancing adaptability and compatibility with immense potential for application.

Epoxy resins and acrylates are two commonly used materials in the protection of wooden artifacts. As a derivative, epoxy acrylate (EA) combines the high reactivity of epoxy groups with the photocuring properties of acrylate groups, making it widely used in high-performance coatings, adhesives, and composite materials [27]. Additionally, EA offers excellent transparency, film-forming ability, and tunable properties. These features make it suitable for rapid curing at interfaces and show great potential in crack reinforcement for artifacts. However, traditional EA suffers from issues such as high rigidity, low hygroscopicity, and limited affinity with wood surfaces, which restrict its long-term stability for long-term crack reinforcement in museum wooden artifacts [28]. The introduction of lignin with multiple hydroxyl groups holds promise for improving the hygroscopic properties of the material. Currently, research on lignin-grafted epoxy acrylates primarily focuses on green synthesis strategies and the enhancement of photopolymerization performance, while systematic studies on their hygroscopic behavior remain relatively scarce. To address these issues, a novel epoxy acrylate (LEA) grafted with dealkalized lignin was designed and synthesized. The material retains the photosensitive groups of EA and incorporates hygroscopic functional molecules through nucleophilic ring-opening reactions with lignin structural units. Analysis results show that lignin modification significantly enhances the hygroscopic performance of epoxy acrylate while achieving mechanical compatibility with artificially degraded wood specimens. LEA effectively reinforces micrometer-scale cracks while preserving the original cellular structure of wood cell walls.

## 2. Materials and Methods

### 2.1. Materials

Dealkalized lignin (DL) (Mw = 1513.58 g/mol) was kindly provided by Yien Chemical Technology Co., Ltd. (Shanghai, China). Anhydrous ethanol and sodium hydroxide (NaOH) were purchased from Macklin Chemical Reagent Co., Ltd. (Shanghai, China). Bisphenol A epoxy acrylate (BPAEA) (average Mw = 500 g/mol; viscosity at 50 °C: 11,000–21,000 mPa·s) was obtained from Covestro Co., Ltd. (Leverkusen, Germany). Imidazole (F.W. = 68.08, AR) was purchased from Sinopharm Chemical Reagent Co., Ltd. (Shanghai, China). The reactive diluent 692 was benzyl glycidyl ether, supplied by Rainbow Stone Composite Materials Co., Ltd. (Suzhou, China). The diluent was supplied by Rainbow Stone Composite Materials Co., Ltd. (Suzhou, China). The photoinitiator 6976 was purchased from Yinchang Chemical Co., Ltd. (Shanghai, China). All chemicals were used as received unless otherwise stated.

Pearwood samples were obtained from Zhongding Cultural Development Co., Ltd. (Shandong, China).

### 2.2. Methods

First, appropriate amounts of DL were dissolved in anhydrous ethanol (0.01 M); the pH value of this solution was adjusted to pH 8 with NaOH, heated at 50 °C, and stirred at 400 rpm for 12 h. After filtration, a dispersed lignin solution was yielded by removing insoluble impurities. Then, 0.3 g of Imidazole (catalyst) and 30 g of BPAEA were added into the above solution and stirred at 70 °C for 4 h. After that, lignin-based epoxy acrylate (LEA) was obtained by removing alcohol from the product through distillation. LEA with different DL concentrations was prepared and tested (Table 1).

### 2.3. Preparation of Blends

**Figure 1:**
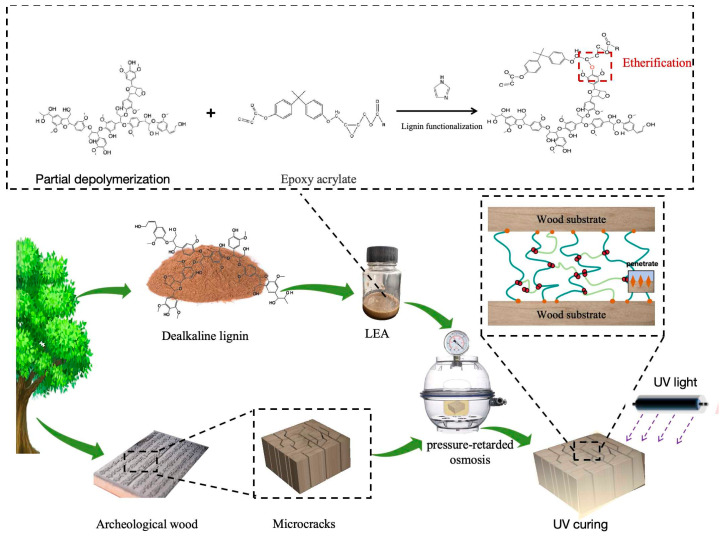
The preparation and curing process for LEA.

The obtained LEAs were blended with photoinitiator 6976 (1 wt%) and diluent 692 (22 wt%) at ambient temperature, and the mixture was stirred for several minutes to ensure complete homogeneous mixing. The viscosity of these blends was around 1500 Pa·s. UV light (T16Pro, Zhejiang shrepa photoelectricity technology) was used to cure the LEA blends. The curing time was 60 s under a wavelength of 365 nm. The power of the UV light was 180 W, and the distance between sample and light bulb was 5 cm. The preparation and curing process is illustrated in Figure 1.

### 2.4. Preparation of Artificially Degraded Wood

Artificially degraded wood was prepared using a hydrothermal alkaline treatment method to evaluate the compatibility between LEA and wooden artifacts [29]. Pearwood samples with dimensions of 1 × 1 × 1 cm were immersed in a 3% (*w*/*v*) NaOH aqueous solution and reacted at 130 °C for 20 h in a high-pressure hydrothermal reactor. After the reaction, the samples were repeatedly rinsed with deionized water until both the wood and the rinse water reached a neutral pH (pH = 7) and were subsequently freeze-dried. The artificially degraded wood exhibited a moisture content of 13.4%, which is comparable to the typical range of 6–12% observed in museum wooden artifacts.

### 2.5. Fourier Transform Infrared Spectrometer

The LEA was characterized using a Fourier infrared spectrometer (Thermo Fisher Nicolet iS 5, Waltham, MA, USA). The LEA samples were coated on KBr window slices, and FT-IR spectra were recorded in the wave number range of 400–4000 cm^−1^ with a scanning resolution of 16 cm^−1^.

A Fourier infrared spectrometer was used to characterize the LEA blends before and after curing. The scanning range was 400–4000 cm^−1^, and the scanning resolution was 16 cm^−1^.

Micro-reflectance Fourier transform infrared (FTIR) analysis was performed on the cured LEA samples. A Thermo Fisher Nicolet iN10 instrument (Thermo Fisher Scientific, Waltham, MA, USA) equipped with a microscope was used for microanalysis. Signal acquisition was carried out using a liquid nitrogen-cooled MCT detector (Thermo Fisher Scientific, Waltham, MA, USA) over the wavenumber range of 4000–650 cm^−1^, with a spectral resolution of 4 cm^−1^ and 128 scans.

### 2.6. Nuclear Magnetic Resonance Spectroscopy

The nuclear magnetic resonance (^1^H NMR and ^13^C NMR) spectra of EA and the synthesized LEA were recorded using a Bruker 400 MHz spectrometer (Bruker Corporation, Billerica, MA, USA) with DMSO-d6 as the solvent for analysis.

### 2.7. Isothermal Hygroscopic Sorption Test

The equilibrium moisture contents (EMCs) of LEA samples at different relative humidity (RH) steps were measured using an SPSx dynamic vapor sorption (DVS). The measurement procedure was set as follows: during the adsorption process at 25 °C, the RH in the sample chamber was gradually increased from 0% to 95%, with increments of 10% per step, followed by a desorption process in which the humidity decreased in the same manner. The equilibrium condition for each sample was set to a mass change of less than 0.001% per minute [30]. The GAB model (Guggenheim–Anderson–de Boer) is a theoretical model commonly used to describe moisture sorption isotherms and is suitable for analyzing multilayer adsorption processes. The DVS curves were fitted using the GAB model, and the equation of the GAB model is the following:

$$EMC=\frac{M_{0}\times C\times K\times RH}{\left(1-K)\times (1-K\times RH+C\times K\times RH\right)}$$

where EMC (g/g) is the equilibrium moisture content; RH (%) is the relative humidity; M0 is the monolayer capacity; C (%) is an equilibrium constant related to monolayer sorption; and K (%) is an equilibrium constant related to multilayer sorption.

Both the LEA and wood were in the form of flat plates, with a diameter-to-thickness ratio of approximately 10:1. The unidirectional moisture diffusion process can be described using the infinite plane sheet model derived from Fick’s second law [31].

$$\frac{M_{t}-M_{0}}{M_{\infty }-M_{0}}=1-\sum_{n=0}^{\infty }{\frac{8}{{-(2n+1)}^{2}\pi^{2}}e}^{\frac{{-(2n+1)}^{2}\pi^{2}D_{eff}}{{4L}^{2}}}$$

Initial condition:

$$\frac{\partial M\left(L,t\right)}{\partial t}=0;M\left(0,t\right)=M_{\infty };M\left(m,0\right)=M_{0}$$

where Mt (g/g) is the moisture content at time t, $M_{\infty }$ (g/g) is the equilibrium moisture content, M0 (g/g) is the initial moisture content, and L is the thickness. The thickness of the samples used in the hygroscopicity experiments was approximately 1 mm. Deff is the effective moisture diffusivity.

### 2.8. Contact Angle Measurement

The contact angle was measured using a JC2000DM (Zhongchen Digital Technology, Shanghai, China). The contact angle value was automatically calculated by the software installed on the computer. The droplet image was captured by a video camera and transmitted to the computer.

### 2.9. Scanning Electron Microscope

Scanning electron microscope (SEM) images of cured LEAs were obtained using a Regulus 8100 cold-field scanning electron microscope (Hitachi High-Tech Corporation, Tokyo, Japan) and surface pore sizes were calculated using Image J 1.8.0. The cured LEA samples were brittle-fractured in liquid nitrogen and coated with a thin layer of gold.

The microstructure of artificially degraded wood reinforced with LEA2 was also examined using SEM after gold coating.

### 2.10. Hygroscopic Expansion Coefficient Testing

The coefficient of linear hygroscopic expansion was measured using a TMA7100 thermomechanical analyzer (Hitachi High-Tech Corporation) coupled with a humidity generator. The tests were conducted in a nitrogen atmosphere over a humidity range of 0% to 90% with a test load of 10 mN. The humidity was maintained at 0% for 480 min, then gradually increased to 90% at a rate of 1.5%/min, and subsequently held at 90% for 960 min. The relative coefficient of hygroscopic expansion (α) was calculated using the following formula:

$$\alpha =\frac{∆L/L_{0}}{∆RH}$$

where ∆L is the change in length, $L_{0}$ is the initial dry length, and ∆RH is the change in relative humidity.

### 2.11. Dynamic Mechanical Analysis

Dynamic mechanical analysis (DMA) was performed using a TA Instruments Q800 analyzer (TA Instruments, New Castle, DE, USA). For tensile mode measurements, LEA specimens (40 × 15 × 1 mm) were prepared and tested at 25 °C under an oscillatory frequency of 1 Hz and a constant strain amplitude of 0.1%. Two specimens from each formulation were measured to ensure reproducibility, the average values were reported. To evaluate the shear modulus of LEA films, a shear-sandwich clamp was installed on the same instrument. The strain-dependent shear storage modulus was recorded at a fixed frequency of 10 Hz during a strain sweep ranging from 0.001% to 1% at 25 °C. The test protocol followed ASTM D4440 [32]. Based on the measured tensile modulus E and shear modulus G, Poisson’s ratio ν was calculated using the classical elasticity relationship for isotropic linear elastic materials:

$$\nu =\frac{E}{2G}-1$$

## 3. Results and Discussion

### 3.1. LEA Characterization

**Figure 2:**
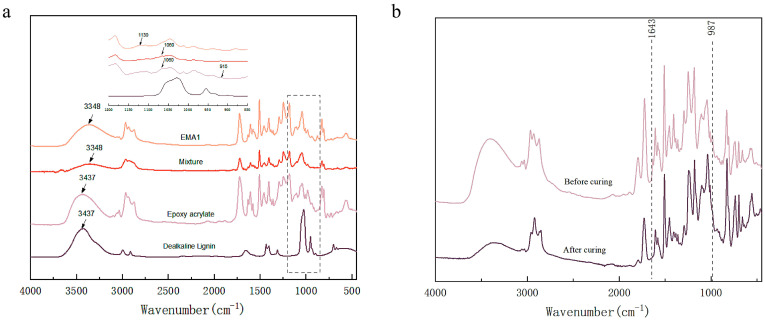
FT-IR spectra of powder (DL) and gel (EA, mixture, LEA2) (**a**); FT-IR spectra of blend and cured product (**b**).

To investigate the reaction mechanism between DL and EA, Fourier transform infrared spectroscopy (FT-IR) was used to monitor the changes in functional groups. The FT-IR spectra of the EA, DL, mixture, and LEA2 are presented in Figure 2a. Both the DL and EA exhibited a characteristic peak at 3437 cm^−1^, corresponding to the O–H stretching vibration. After mixing, this peak shifted to lower wavenumbers, indicating the formation of hydrogen bonds between the DL and EA. The EA showed characteristic epoxy absorption peaks at 1060 cm^−1^ and 915 cm^−1^ [33]. After the reaction, these peaks disappeared, and a new peak appeared at 1130 cm^−1^, assigned to the secondary hydroxyl groups [34]. The changes in characteristic peaks before and after curing are shown in Figure 2b. The peaks at 987 cm^−1^ and 1643 cm^−1^, corresponding to the C=C bonds of the acrylic ester, completely disappeared after curing, demonstrating that the photocurable groups were acrylic double bonds. These characteristic peak changes were consistent with the formation of the LEA.

**Figure 3:**
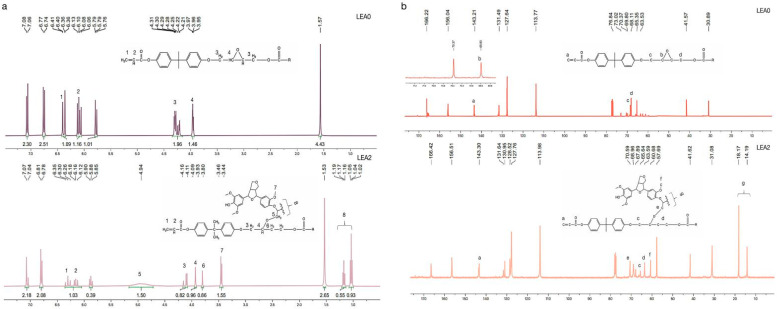
^1^H NMR spectra of LEA0 and LEA2 (**a**); ^13^C NMR spectra of LEA0 and LEA2 (**b**).

The structural characteristics and synthesis of LEA were confirmed by ^1^H NMR and ^13^C NMR. As shown in the ^1^H NMR spectrum (Figure 3a), the proton signals corresponding to acrylate double bonds (δ 6.39–6.04 ppm) still present in the LEA2, indicating that the double bonds remained unreacted during the reaction. This suggests that the photosensitive groups were preserved for subsequent photopolymerization. Consequently, the characteristic epoxy methylene peaks (δ 4.35–4.15 ppm) completely disappeared in LEA2, while a new singlet appeared at δ 4.94 ppm, assigned to the methylene group (–O–CH_2_–) formed through nucleophilic ring-opening reaction between lignin hydroxyl groups and epoxy groups [35].

The ^13^C NMR spectrum (Figure 3b) further supports this conclusion. The retention of the carbon signal at δ 143.30 ppm, corresponding to the double bond, confirms the structural integrity of the acrylate segment. A new characteristic peak at δ 70.59 ppm, attributed to ether-bonded methylene carbons (–O–CH_2_–O–), provides evidence of selective epoxy ring-opening. In addition, characteristic signals of lignin such as the methoxy group (δ 3.45 ppm) and aliphatic side chains (δ 1.02–1.19 ppm) are also observed in the LEA2 spectrum, indicating that the original structure of lignin remained intact during the grafting process. This reaction mechanism achieved the efficient grafting of DL onto EA, introducing lignin-based units, thereby enhancing compatibility between EA and archeological wood.

**Figure 4:**
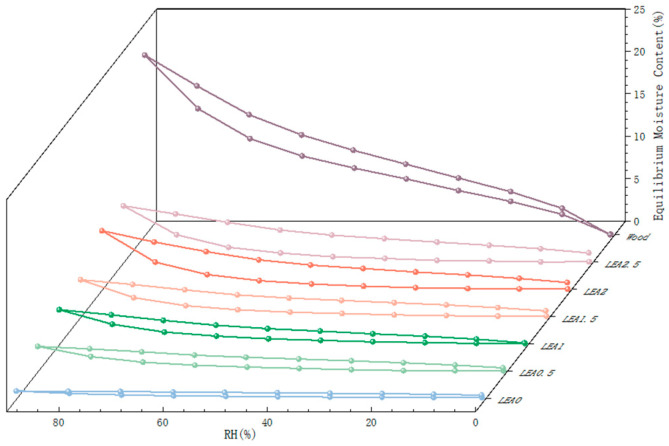
Moisture sorption isotherms and EMC decrease rates of artificially degraded wood and LEA samples.

To ensure the long-term stability of the consolidation system and mitigate interfacial failure risks induced by hygroscopic mismatch, the hygroscopic properties of artificially degraded wood and LEA samples were evaluated using dynamic vapor sorption (DVS), as shown in Figure 4. The sorption isotherms were fitted using MATLAB 2023b, and the GAB model parameters along with fitting accuracy (R^2^) are summarized in Table 2. All samples exhibited high coefficients of determination (R^2^ > 0.99), indicating that the GAB model effectively describes the sorption behavior of both artificially degraded wood and LEA samples. In the GAB model, M_0_ represents the monolayer moisture content, C is a constant related to the heat of adsorption for the monolayer, and K is the multilayer adsorption coefficient. The moisture sorption behavior of the artificially degraded wood exhibited significant differences compared to LEA materials. The wood followed a Type II isotherm according to IUPAC (International Union of Pure and Applied Chemistry) classification, with hygroscopic behavior governed by hydroxyl-dominated monolayer adsorption (M_0_ = 4.16 g/g, C = 9.43) and multilayer adsorption (K = 0.84). In contrast, the LEA samples exhibited typical Type III isotherms [36], characterized primarily by multilayer adsorption. The C values of all LEA formulations were below 5.67, suggesting negligible monolayer adsorption and weak hygroscopicity in low-humidity environments [37]. When RH exceeded 40%, the K value of the LEA system increased with higher lignin content, enhancing overall hygroscopic capacity through pronounced multilayer adsorption. Within this humidity range, the LEA and artificially degraded wood exhibited similar sorption behavior. This hygroscopic trend matching under high humidity conditions can reduce the risk of consolidation system failure at the typical relative humidity levels found in museums (50–60% RH). LEA1.5 to LEA2.5 demonstrated distinct desorption hysteresis loops accompanied by capillary condensation effects during adsorption–desorption cycles [38]. These results indicate that lignin grafting optimizes multilayer adsorption capacity, enabling dynamic moisture response matching with archeological wood and effectively alleviating interfacial stress mismatch caused by hygroscopic incompatibility.

**Figure 5:**
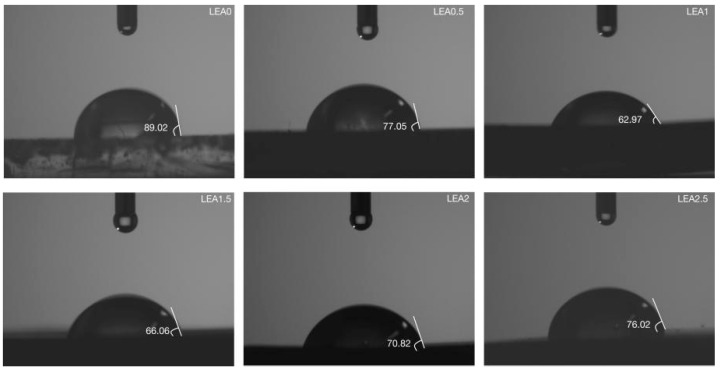
Contact angle of LEA samples.

**Figure 6:**
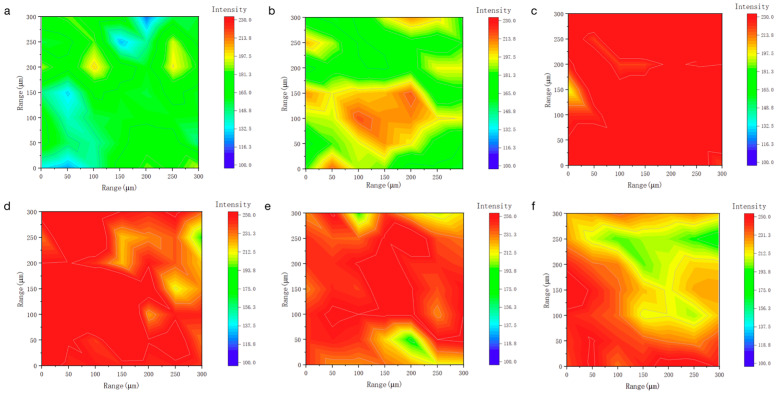
The used LEA maps obtained from the 3570 cm^−1^ spectra that make up the FTIR map: (**a**) LEA0, (**b**) LEA0.5, (**c**) LEA1, (**d**) LEA1.5, (**e**) LEA2, (**f**) LEA2.

**Figure 7:**
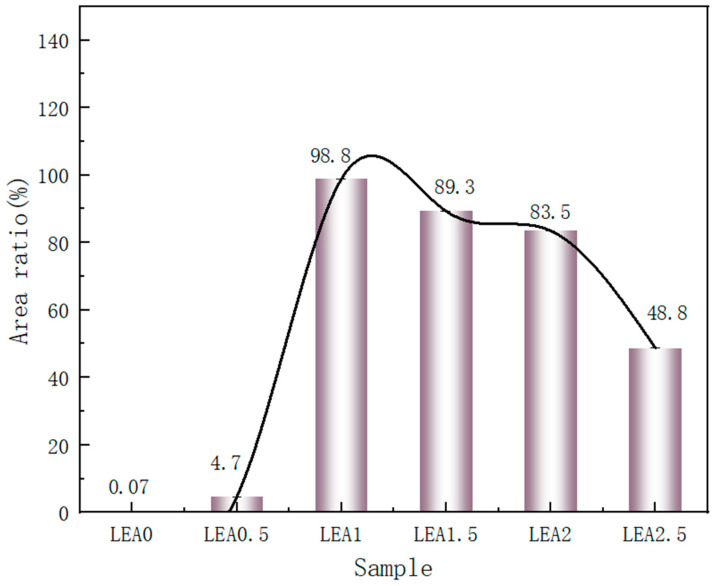
Measuring the red area in each micro-FTIR map.

To investigate the monolayer adsorption ability of LEA, the initial moisture adsorption behavior was analyzed through hydrophilicity and hydroxyl group density. Hydrophilicity facilitates the initial attachment of water molecules and enables diffusion paths for multilayer adsorption. The hydrophilicity of LEA samples with varying lignin grafting levels was measured using a contact angle goniometer, and the results are shown in Figure 5. As the DL content increased, the contact angle first decreased and then increased, with LEA1 exhibiting the smallest angle and indicating the strongest hydrophilicity. The hydrophilic behavior of LEA mainly originates from hydroxyl groups in DL. To analyze hydroxyl distribution and density, micro-FTIR surface mapping was conducted using the free hydroxyl characteristic peak at 3570 cm^−1^. The resulting FTIR maps are shown in Figure 6. By quantifying the red areas, the surface hydroxyl density of each sample was determined (Figure 7). Hydroxyl density initially increased with lignin grafting, peaking in LEA1 with uniform distribution, and then decreased at higher grafting levels. Excessive lignin grafting induced lignin aggregation, where internal hydrogen bonding reduced free hydroxyl sites. This trend aligned with M_0_ variations in the GAB model, confirming that surface-exposed free hydroxyl groups critically regulate monolayer adsorption. Higher hydrophilicity and hydroxyl density facilitate faster surface wetting and the diffusion of water molecules on LEA, thereby enhancing the material’s responsiveness to ambient humidity fluctuations. This contributes to dynamic coordination between LEA and aged wood during the initial moisture adsorption stage.

**Figure 8:**
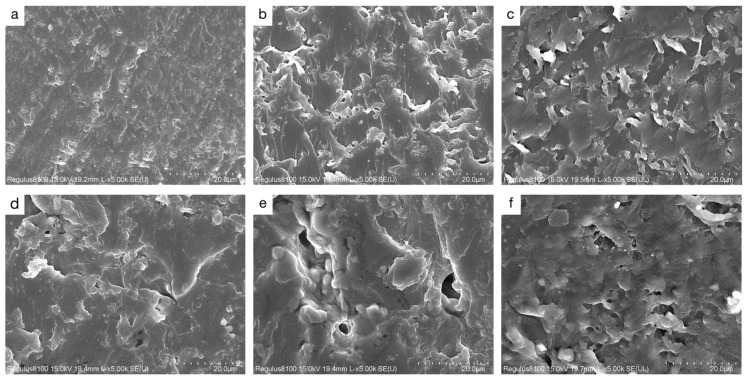
SEM image of LEA samples: (**a**) LEA0, (**b**) LEA0.5, (**c**) LEA1, (**d**) LEA1.5, (**e**) LEA2, (**f**) LEA2.5.

**Figure 9:**
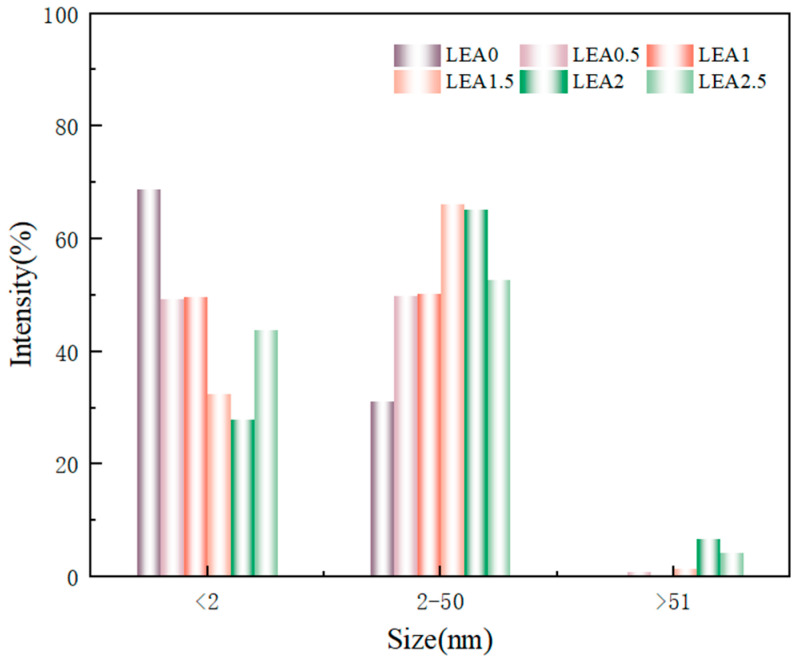
Pore size distribution of LEA samples.

The microstructure of a material significantly influences its moisture adsorption performance. To investigate the pore structure of the LEA samples, SEM was used to observe the fracture surfaces (Figure 8). Image J software was employed to statistically analyze the surface pore size distribution, and the results are shown in Figure 9. The LEA0 sample exhibited a dense and smooth surface with very few pores. Combined with the pore size distribution results, it was found that LEA0 mainly consisted of micropores (<2 nm), which limited the adsorption and diffusion of water molecules. As the lignin grafting contents increased, SEM images (Figure 8b,e) revealed that the samples gradually developed interconnected porous network structures. The proportion of mesopores (2–50 nm) increased progressively, and in samples LEA1.5 to LEA2.5, mesopores became the dominant pore type, surpassing micropores. Such pore structures not only facilitated water molecule adsorption on pore walls but also provided sufficient space for capillary condensation, leading to noticeable desorption hysteresis. LEA2 exhibited the highest mesopore content, reaching 66.2%. Combined with moisture sorption curves, it can be concluded that mesoporous structures promote water accumulation and transport within the pore channels, enhancing the multilayer adsorption capacity of the material. However, when the lignin content increased to LEA2.5, SEM images (Figure 8f) showed that excessive DL partially covered the pore surfaces, resulting in structural blockage. Consequently, the proportion of micropores increased while mesopores decreased, which reduced the overall moisture sorption capacity. This trend was consistent with the variation in the K value from the GAB model, confirming that an appropriate amount of lignin grafting promotes mesopore formation. The presence of mesopores plays a critical role in enhancing the multilayer adsorption capability of LEA, thereby improving its overall hygroscopicity.

**Figure 10:**
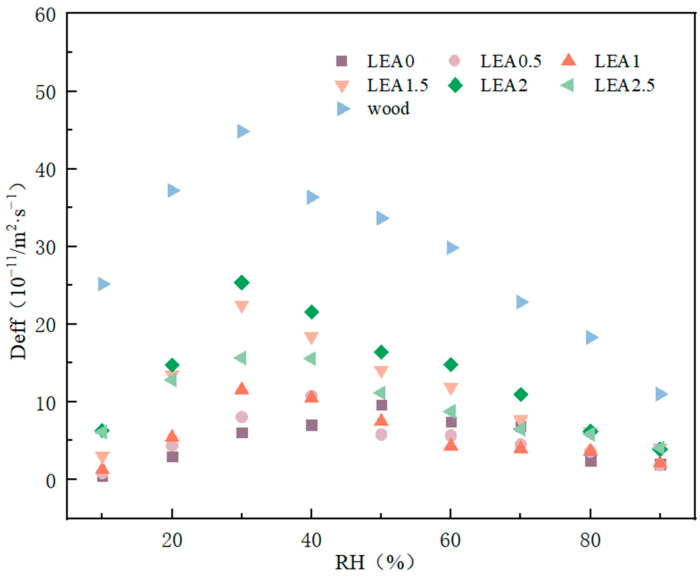
Effective moisture diffusivity with different RH.

The effective moisture diffusivity (Deff) reflects the ability of water molecules to migrate within a material and serves as a key parameter for characterizing the moisture sorption rate. Based on Fick’s second law, the Deff values of different LEA samples under various RH conditions were calculated, and the results are shown in Figure 10. The relationship between lignin grafting content and Deff was nonlinear; as the lignin content increased from LEA0 to LEA2, the diffusion coefficient increased accordingly. LEA2 exhibited excellent moisture diffusion performance across all humidity ranges, indicating that an appropriate amount of lignin grafting effectively enhances water diffusion efficiency in humid environments. However, excessive lignin grafting reduced mesopore content, leading to decreased Deff. In addition, Deff showed a characteristic trend of initially increasing and then decreasing with rising RH. At low RH levels, water primarily diffused into the material through pores. When RH exceeded a critical point, Deff decreased, possibly due to the moisture-induced swelling of the internal polymer network, which limited further water diffusion. Deff also exhibited distinct humidity responsiveness, with peak values occurring at different RH levels. For LEA0, the maximum Deff was observed near 50% RH, while for lignin-grafted samples, the peak shifted toward lower humidity. Specifically, the Deff of LEA1 to LEA2.5 reached its maximum at around 30% RH, which corresponds well with the humidity-responsive behavior of artificially degraded wood. These results indicate that LEA materials can achieve synchronized hygroscopic swelling with artificially degraded wood under varying humidity, contributing to improved compatibility between the consolidant and the wooden substrate.

**Figure 11:**
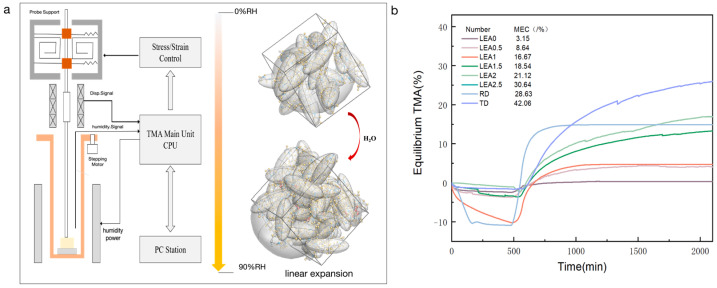
(**a**) Moisture swelling schematic of LEA samples. (**b**) Relative hygroscopic expansion coefficient of LEA samples and artificially degraded wood.

The crosslinking density of a polymer network plays a critical role in moisture sorption behavior, manifested in moisture-induced swelling. To quantitatively characterize humidity-responsive dimensional changes, hygroscopic expansion behaviors were evaluated using a TMA coupled with a humidity generator. As the RH increased from 0% to 90%, water molecules gradually penetrated the crosslinked network, inducing polymer chain mobility and volumetric expansion, as illustrated in Figure 11a. The dimensional changes in the samples under controlled humidity are presented in Figure 11b. With increasing lignin grafting content, the relative HEC of the LEA samples showed a significant rise. Compared to LEA0, LEA2.5 exhibited nearly a tenfold increase in the expansion coefficient, indicating that lignin grafting markedly enhanced the flexibility and humidity-responsiveness of the polymer network. Notably, the HECs of LEA2 and LEA2.5 closely matched the radial expansion behavior of aged wood, suggesting their potential for synchronized deformation with wooden artifacts.

In combination with the Deff results (Figure 10), it is evident that lignin grafting not only improves the water diffusion rate of LEA materials in low-humidity environments but also endows them with humidity-responsive dimensional adjustment under high-RH conditions. This synchronous and amplitude-matched hygroexpansion behavior can effectively mitigate interfacial stress accumulation caused by humidity fluctuations, thereby enhancing the mechanical compatibility and long-term stability of the consolidation system.

Significant differences in the elastic modulus (E) and Poisson’s ratio (ν) between the consolidant and the wooden substrate can lead to interfacial stress accumulation, compromising the stability of the reinforcement system. To evaluate the mechanical compatibility of the LEA, dynamic mechanical analysis (DMA) was performed to measure the elastic modulus (E’), and the results are presented in Table 3. As the lignin grafting content increased, the elastic modulus of LEA decreased markedly from 350 MPa (LEA0) to 3.8 MPa (LEA2.5). This trend is attributed to the plasticizing effect of lignin and the steric hindrance of its aromatic structure, which disrupted the crosslinked network formed during photopolymerization. Compared with the tensile modulus of artificially degraded wood in the radial direction (approximately 276 MPa), LEA0 exhibited evident stiffness mismatch. Under fluctuating humidity conditions, such mismatch can generate considerable interfacial stress, accelerating substrate damage. In contrast, lignin-grafted LEA showed a substantial reduction in the modulus and exhibited pronounced viscoelasticity, which helped alleviate interfacial stress caused by rigidity mismatch.

Moreover, Poisson’s ratio is another key parameter for evaluating mechanical compatibility and interfacial stress response. Based on the elastic modulus (E’), shear modulus (G’), and loss modulus (G’’), Poisson’s ratios of the LEA samples were calculated using the bulk modulus method. The Poisson ratios of LEA0.5 to LEA2 ranged from 0.39 to 0.35, close to that of artificially degraded wood (≈0.38), indicating good compatibility in transverse deformation. Therefore, by tailoring the elastic modulus, shear modulus, and Poisson ratio through controlled lignin grafting, it is possible to reduce interfacial stress between LEA and the wooden substrate, thereby improving the long-term stability and mechanical durability of the reinforcement system.

### 3.2. Application Properties

**Figure 12:**
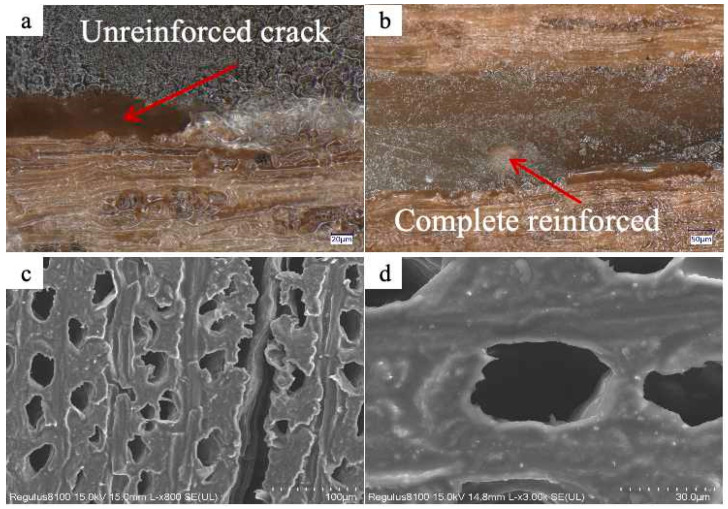
(**a**) Microcrack reinforcing in artificially degraded wood by LEA0. (**b**) Microcrack reinforcing in artificially degraded wood by LEA2. (**c**) SEM image of wood vessels after consolidation with LEA2. (**d**) SEM image of wood cell walls after consolidation with LEA2.

To assess the efficacy of LEA materials in reinforcing microcracks within artificially degraded wood, LEA0 and LEA2 were, respectively, formulated with diluent 692 and photoinitiator 6976 at a mass ratio of 10:30:5. The blends were thoroughly stirred to ensure homogeneity. These blends were then used to infiltrate wood with an average crack width of 260 μm, and the reinforcing effect is shown in Figure 12. After treatment with LEA0, unreinforced regions were still observed within the wood cracks. In contrast, samples treated with LEA2 showed the uniform and thorough reinforcement of microcracks. The lignin modification significantly improved the interfacial affinity between EA and the wood. SEM images revealed that the LEA2 blend was distributed exclusively within the cracks, without penetrating into vessel lumens or cell walls, thus preserving the original microstructure of the wood. The LEA material demonstrated excellent selective reinforcing capability, contributing to the reinforcement of localized structural integrity while avoiding excessive interference with the wood artifacts. These results highlight the promising application potential of LEA materials in the conservation of aged wooden artifacts.

## 4. Conclusions

To address the issues of interface failure and mechanical incompatibility caused by the hygroscopicity differences between reinforcement materials and museum wooden artifacts under fluctuating humidity conditions, LEA was synthesized. A secondary hydroxyl peak appeared at 1130 cm^−1^ in the FT-IR spectrum. In addition, characteristic signals were observed at δ 4.94 ppm in the ^1^H NMR spectrum and 70.59 ppm in the ^13^C NMR spectrum. These signals are attributed to methylene groups formed via nucleophilic ring-opening reactions between lignin hydroxyl groups and epoxy moieties, collectively confirming the successful grafting reaction between dealkalized lignin and epoxy acrylate. After lignin grafting, the hygroscopic performance was significantly enhanced, with the relative humidity-induced swelling coefficient increasing tenfold. The results showed that the LEA2 sample exhibited excellent multilayer adsorption capability. It also demonstrated the potential for synchronous and amplitude-matched hygroscopic expansion similar to that of archeological wood. DMA revealed that LEA2 had an elastic modulus of 261.58 MPa and a Poisson’s ratio of 0.35, comparable to those of artificially degraded wood. The material effectively alleviated the interface stress caused by rigidity differences, enhancing the long-term stability and durability of the reinforcement system. LEA2 effectively reinforced wood cracks, strengthened local structural stability, and preserved the microstructure of the wood. This study is the first to systematically integrate hygroscopic regulation with mechanical compatibility through lignin-grafted epoxy acrylate, providing a novel approach for environmentally responsive crack reinforcement. LEA holds great potential in the reinforcement of museum wooden artifacts. With excellent hygroscopicity and mechanical compatibility, LEA maintains stability in fluctuating humidity environments, significantly enhancing the long-term stability of the reinforcement system and the effectiveness of cultural relic protection.

## Figures and Tables

**Table 1:** Composition of LEA.

Number	DL (g)	Ethanol (mL)	Imidazole (g)	BPAEA (g)
LEA0	0	0	0	30
LEA1	1.0	78.5	0.3	30
LEA1.5	1.5	117.8	0.3	30
LEA2	2.0	157	0.3	30
LEA2.5	2.5	196.2	0.3	30

**Table 2:** Fitting parameters and evaluation indexes of GAB sorption models.

	Wood	LEA0	LEA0.5	LEA1	LEA1.5	LEA2	LEA2.5
M_0_	4.16	0.65	1.43	1.52	1.08	0.96	4.86
C	9.43	0.17	0.29	0.35	0.39	0.81	0.90
K	0.84	0.63	0.84	0.87	0.93	0.98	0.87
R^2^	0.99	0.99	0.99	0.99	0.99	0.99	0.99

**Table 3:** DMA mechanical performance test results of LEA samples.

	Elastic Modulus (MPa)	Shear Modulus (MPa)	Loss Modulus (MPa)	Poisson’s Ratio
LEA0	350.02	136.11	25.59	0.29
LEA0.5	314.72	113.21	25.49	0.39
LEA1	285.33	102.64	26.19	0.39
LEA1.5	268.92	98.87	26.11	0.36
LEA2	261.58	96.88	27.25	0.35
LEA2.5	189.86	65.47	30.65	0.45

## Data Availability

Data are contained within the article.
